# Odour Samples Degradation During Detention in Tedlar^®^ Bags

**DOI:** 10.1007/s11270-015-2495-2

**Published:** 2015-06-30

**Authors:** Mirosław Szyłak-Szydłowski

**Affiliations:** Faculty of Environmental Engineering, Warsaw University of Technology, Warsaw, Poland

**Keywords:** Chromatography, Containers, Odours, Sampling, Tedlar

## Abstract

In indirect olfactometry analysis, to avoid condensation or adsorption processes during or storage of the sample, containers made of suitable materials should be used. Also, reaction between the chemicals during transport from the source of the odour to the research laboratory is an important process which can influence on examinations’ results. Study included determination of the odour and compound concentrations of six gas mixtures. Gas samples were collected by silicone hoses into Tedlar^®^ bags and tested by Nasal Ranger, SM-100 olfactometers and Photovac Voyager gas chromatograph. Time of keeping gas in bags was 78 h, and concentration of compounds was measured every hour, eight times per day. For benzene, acetone, 1,1-dichloroethylene, c-1,2-dichloroethylene, t-1,2-dichloroethylene, methyl ethyl ketone and vinyl chloride, 100 % decrease of concentration has been noticed within 78 h of holding in the bag. Average rate of loss of most compounds concentration was from 0.01 to 2.50 % for the first 30 h and from 0.35 to 18.50 % during the last 48 h of examination. Decreasing of odour concentration measured by Nasal Ranger (NR) in all series was between 0.00 and 4.98 % till 30 h, between 1.91 and 100 % in the last 48 h of test and between 1.61 and 100 % in 78 h. In case of odour concentration measured by SM, those values were, respectively, 1.26–4.93 %, 1.39–4.93 % and 2.40–3.18 %. Values of average rate of intensity decreasing were, respectively, 0.77–1.75 %, 2.36–4.67 % and 1.18–2.07 %. Statistically significant correlation coefficients for compound concentrations and intensity, odour concentration obtained by SM-100 as well as NR were, respectively, 0.55–0.97, 0.47–0.99 and 0.37–0.98.

## Introduction

Zarra et al. (2010) mention two research directions used for studies of odour impact: measurement of odour emissions from stationary sources (methods: sensory analytical and instrumental) and an assessment of the impact of the whole area (methods: modeling dispersion in the air—this method requires the definition and analyze multiple input parameters, which are not always available—and field measurement techniques). Olfactometry research can be divided into indirect and direct olfactometry. Direct (field) olfactometry is based on the analysis of the air directly from the source, while in the case of indirect olfactometry, the sample must be pressed into a suitable container and, in a second stage, be analyzed. Achieved results vary widely between laboratories (Schulz and van Harreveld 1996) and that human factors affect sensory panel performance (Bliss et al. 1996). Also, to avoid condensation or adsorption processes during or storage of the sample, containers made of suitable materials should be used. The primary advantage of direct olfactometry is to minimize both the above mentioned phenomena, as well as the reaction between the chemicals during transport from the source of the odour to the research laboratory. If we consider the olfactometry analysis, which requires the participation of an expert panel, direct olfactometry has the disadvantage that it is very expensive due to the need for researcher’s departure to the site of odour event. Furthermore, there is a risk that the presence of test persons at the site can affect their responses—on the one hand due to the origin of the sample consciousness, on the other hand, the possibility of impact from odours in the background. Stuetz et al. (1998) mentioned that an instrument that mimics the response given by a sensory panel may offer more repeatable and reproducible results. Unfortunately, use of various sensory technologies developed and used in food industry to discriminate between varying levels of odours (Hodgins 1995) is limited in environmental odour measurements. Field olfactometers make a series of dilutions by mixing the stream of fragrant air flow with stream of air filtered through a carbon filter. In the field olfactometry, measurement result is the degree of fragrant gas dilution, set by sniffing person at the time that the gas is at the threshold of sensibility. It is recorded as a ratio of the mixed gas stream D/T (dilution-to-threshold). One of the most commonly used field olfactometers are as follows: Nasal Ranger Field Olfactometer (St. Croix Sensory, Inc.) and SM-110 (IDES Canada Inc.).

These olfactometers are used for direct olfactometry field techniques, partly modeled on EN 13725. Many researchers, including Pan et al. (2007), Nicell (2009), Trabue et al. (2011) and Wang et al. (2011) point to the fact that this method is one of the most frequently used during analysis in the field, while Etievant (2008) describes the use of its application to the study of pathology of smell. Bokowa (2012) compared the odour concentrations measured by olfactometer Nasal Ranger, dynamic olfactometry (DDO) and portable olfactometer Scentroid SM 110, one of the modern tools for olfactometric measurements. She showed a good correlation between the results obtained using the olfactometer SM 110 and DDO but found a 38 % difference between the results of odour concentration within the range of 2000–4000 ou. The results obtained using the Nasal Ranger olfactometer were significantly lower than the others. Henry et al. (2011), comparing the results obtained, among others, Nasal Ranger, DTFCO triangle test and Odor Intensity Scale Reference (OIRS), analyzed the D/T ratio of between 0 and 60. Trabue et al. (2011) characterized the disadvantages and limitations of olfaction dynamic methods, including changes in the composition of the odorants during sample transport to the laboratory (Traube et al. 2006) as well as errors in the air intake into the bags. Additionally, Davoli et al. (2012) drew attention to the potentially toxic compounds (PTC), which could be dangerous for exposed using the DDO method. The current European standard imposes a 30-h expiration period on all samples after which odour analysis cannot be performed (Bakhtari 2014).

The main goal of the study was to determine the uncertainty in measurement when chosen samples have been stored for up to 24 h. The data is detailed in determining which chemicals work better for long-term storage in Tedlar^®^ bags. Also, differences in the results obtained using two olfactometers SM-100 and Nasal Ranger had been examined.

## Materials and Methods

The study included determination of the odour concentrations of six gas mixtures using the Nasal Ranger and Scentroid SM 100 olfactometers. Furthermore, the odour intensity was determined on a scale from 0 (no odour) to 6 (extremely strong) according to Richtlinien VDI 3882 (VDI 2008) . The gas samples were collected by silicone hoses into polyvinyl fluoride (PVF, brand name Tedlar^®^) bags and be tested by field olfactometers. Concentration of the compounds was determined using a Photovac Voyager gas chromatograph. Time of keeping gas in the Tedlar^®^ bags was 78 h, and the concentration of compounds was measured every hour, eight times per day.

Nasal Ranger olfactometer is a lightweight, portable instrument with two built-in replaceable filter cartridges with activated carbon for air purification to the state of odourless. It contains embedded system channels for mixing and dividing gas streams—consciously targeting known part of the inhaled air by avoiding the filters. The control valve is used to adjust one of the 11 values of D/T (2, 4, 7, 15, 30, 60, 100, 200, 300, 400, 500) and to set the “blank”, at which the researcher breathes by purified air stream. During measurement with the Scentroid SM 100 olfactometer, the polluted air is diluted by the technical air pumped from the tank. Scentroid patented valve allows to set it at 15 positions corresponding to the 15 values of D/T. The removable plates with holes of different diameters allows for determination of D/T in the range 2–30,000. During the measurement, as in laboratory indirect olfactometry, researchers increase the value of the D/T, to achieve the individual odour threshold.

Photovac Voyager is a portable, automatic gas analyzer for identifying airborne chemicals and measuring their concentrations. Voyager uses a gas chromatograph (GC) to analyze air samples and collects a sample of air and automatically introduces the sample into the GC. It uses photoionization detector (PID), precolumn and three columns for heavy (C7-C12), middle (C3-C7) and light (C1-C3) compounds): 4 m × 0.53 mm × 2.0 um SPB-35 (precolumn), 8 m × 0.25 mm BLANK Fused Silica (column A), 20 m × 0.32 mm × 1.0 um Supelcowax10 (PEG) (column B), 15 m × 0.32 mm × 12 um Quadrex 007–1 (column C). Carrier gas used for determinations was a high purity nitrogen. Column oven in Photovac Voyager is isothermal 55 to 80 °C. The Voyager can be effectively used to monitor many of the volatile organic compounds listed in EPA Method 8240A (EPA 1996b), including chlorinated and aromatic hydrocarbons. Method detection limits (MDLs) for VOCs range from parts per trillion (ppt) in water (ng/L) to about 500 parts per million (ppm) in ambient air, depending upon the type of compound and detector used (EPA/600 1998).

Tedlar^®^ gas sampling bags of 50 μm thickness used for determinations are recommended in many US EPA methods including TCLP and methods TO-3 (VOCs), TO-12 (NMOC), TO-14A (VOCs), TO-15 (VOCs), ASTM D-5504 (reduced sulfur compounds) and a variety of atmospheric gas methods (Sigma aldrith 2014). It is the predominant material used for sampling bags in the USA, while Nalophan is more widely used in Europe and Australia (Parker et al. 2010). Inter alia, Kim et al. (2013) used this material (10 l bags), samples analyzed within 12 h, to diagnostic analysis of offensive odorants in a large municipal waste treatment plant in an urban area. Bags used in research were equipped with dual stainless steel fittings. The samples were stored at room temperature (21.5 °C) and weren’t subjected to sunlight or any UV emitting artificial lighting. Reported background values without pre-cleaning were in the range 20–60 OU in Tedlar, 30–100 in Nalophan (Parker et al. 2003, Juarez-Galan et al. 2008). Miller and McGinley (2008) found that preconditioning Tedlar bags by flushing with odour free air at an elevated temperature reduced odour background levels to within 2× the reporting limit. Also, Alvarado et al. (2015) during hydrogen sulfide and ammonium determination, prior to gas collection purged the Tedlar bags with clean air (zero gas) twice. According to that, bags were flushed twice with odour free air from tank used for olfactometry examinations. External relative humidity was 60 % and the temperature was 21 °C.

The data from laboratory experiments were subjected to statistical analysis using R 3.1.1. environment and Statsoft Statistica 10 software.

## Results and Discussion

During research, 16 compounds were identified (Table 1).

**Table 1:** Compounds identified during research

Name of the compound (abbrev.)			
Acetone (act)	Benzene (ben)	Bromomethane (brm)	Carbon disulfide (cs2)
Chlorobenzene (clb)	Chloroethane (cle)	1,1-dichloroethylene (1 cl)	c-1,2-dichloroethylene (c12)
t-1,2-dichloroethylene (t12)	2-hexanone (2hx)	Methyl ethyl ketone (mek)	Trichloroethylene (3et)
Tetrachloroethylene (4ce)	Vinyl acetate (vac)	Vinyl chloride (vcl)	m-xylene (mxy)

Abbreviations in brackets are abbreviations used in text

**Fig. 1:**
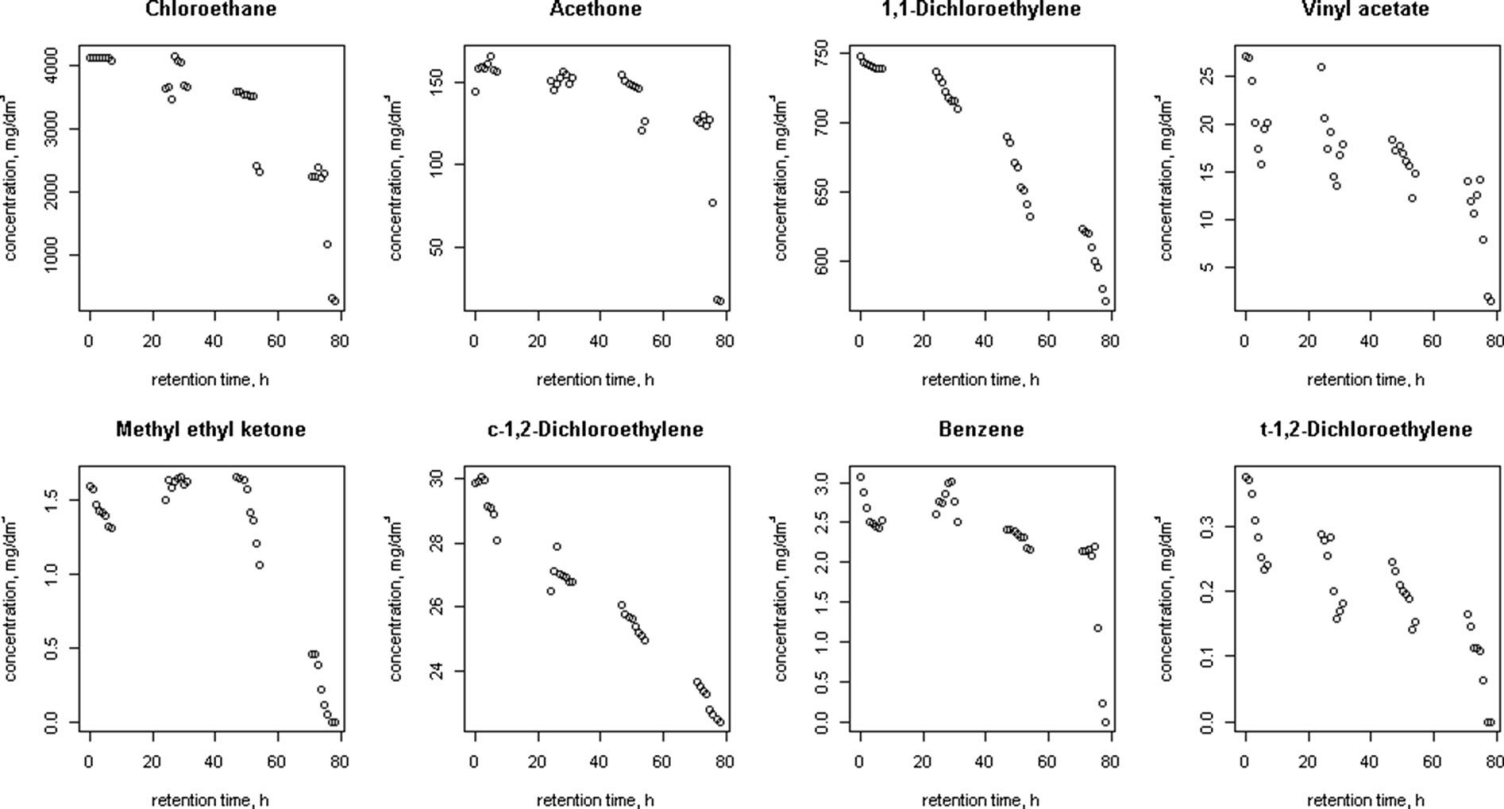
Changes in the concentration of compounds during 78 h of detention in a Tedlar^®^ bags; series 1

**Fig. 2:**
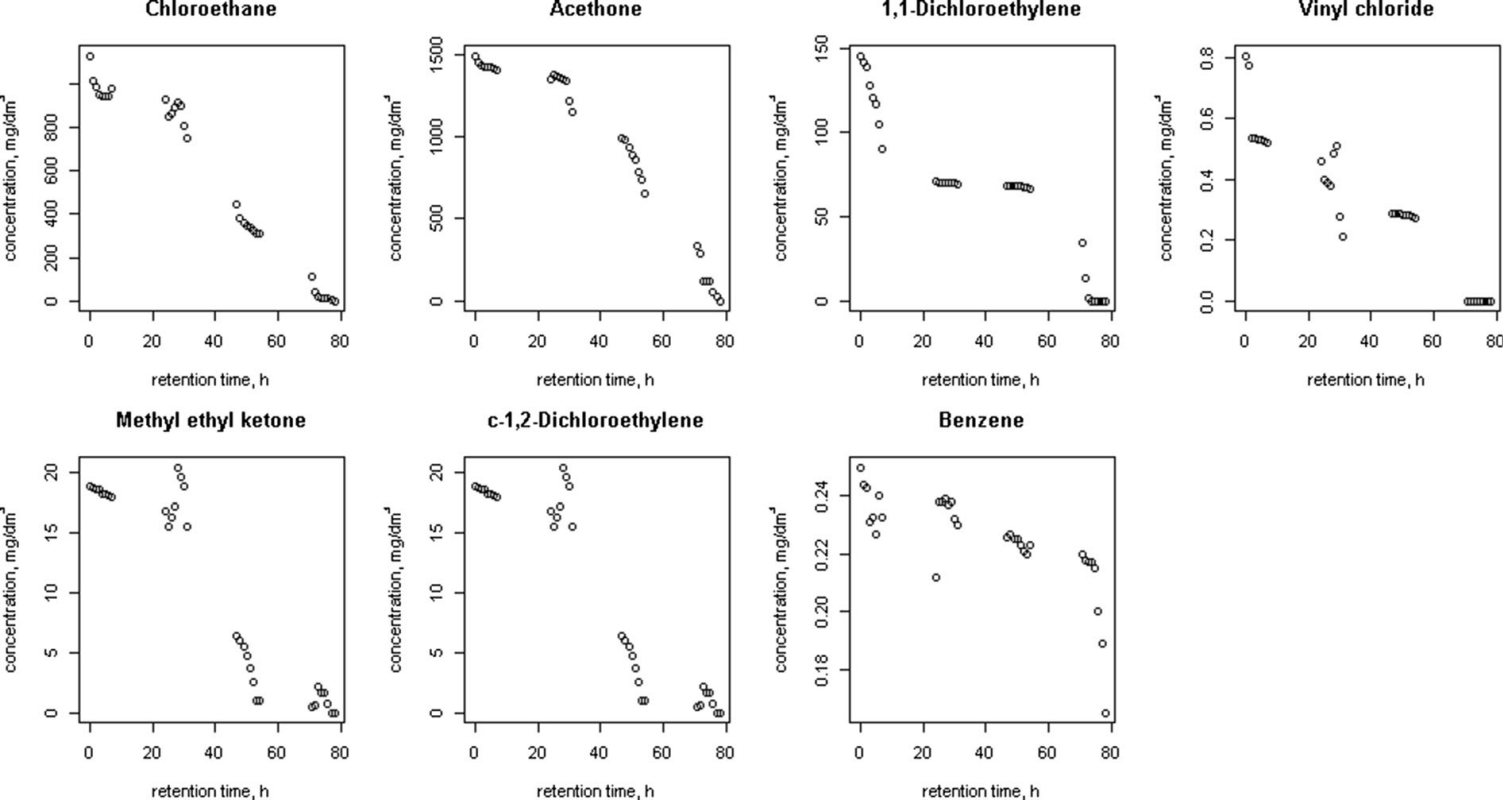
Changes in the concentration of compounds during 78 h of detention in a Tedlar^®^ bags; series 2

**Fig. 3:**
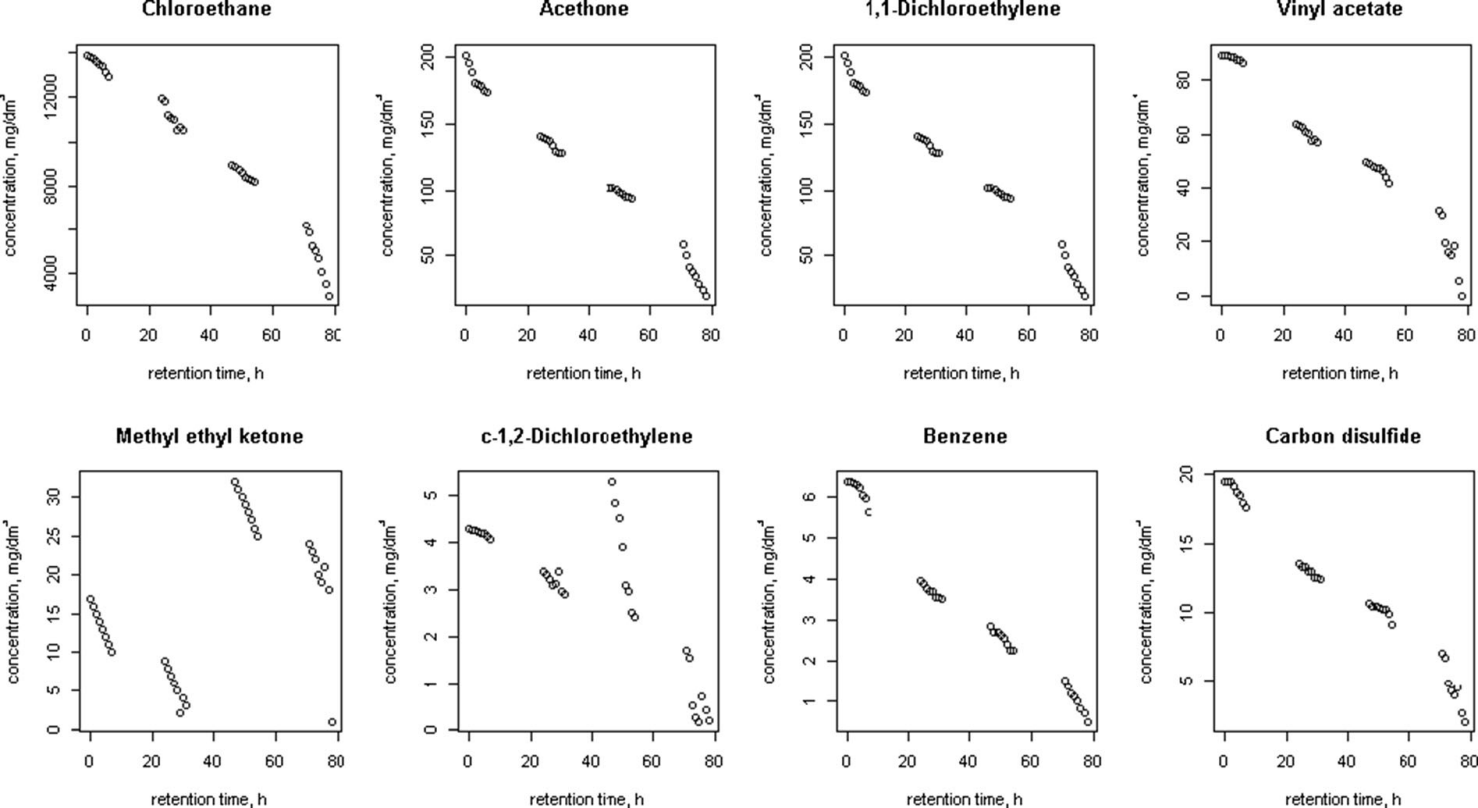
Changes in the concentration of compounds during 78 h of detention in a Tedlar^®^ bags; series 3

**Fig. 4:**
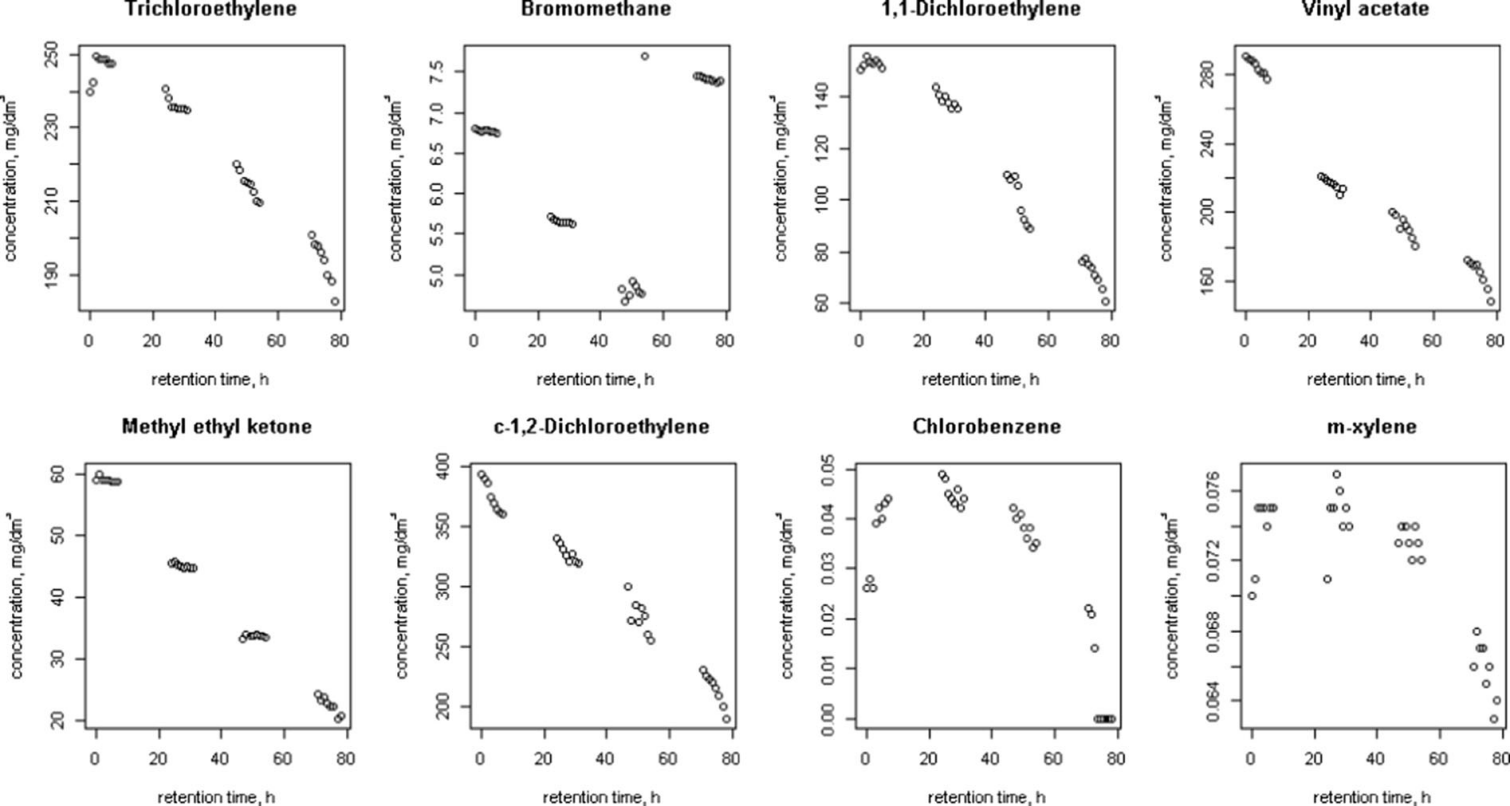
Changes in the concentration of compounds during 78 h of detention in a Tedlar^®^ bags; series 4

**Fig. 5:**
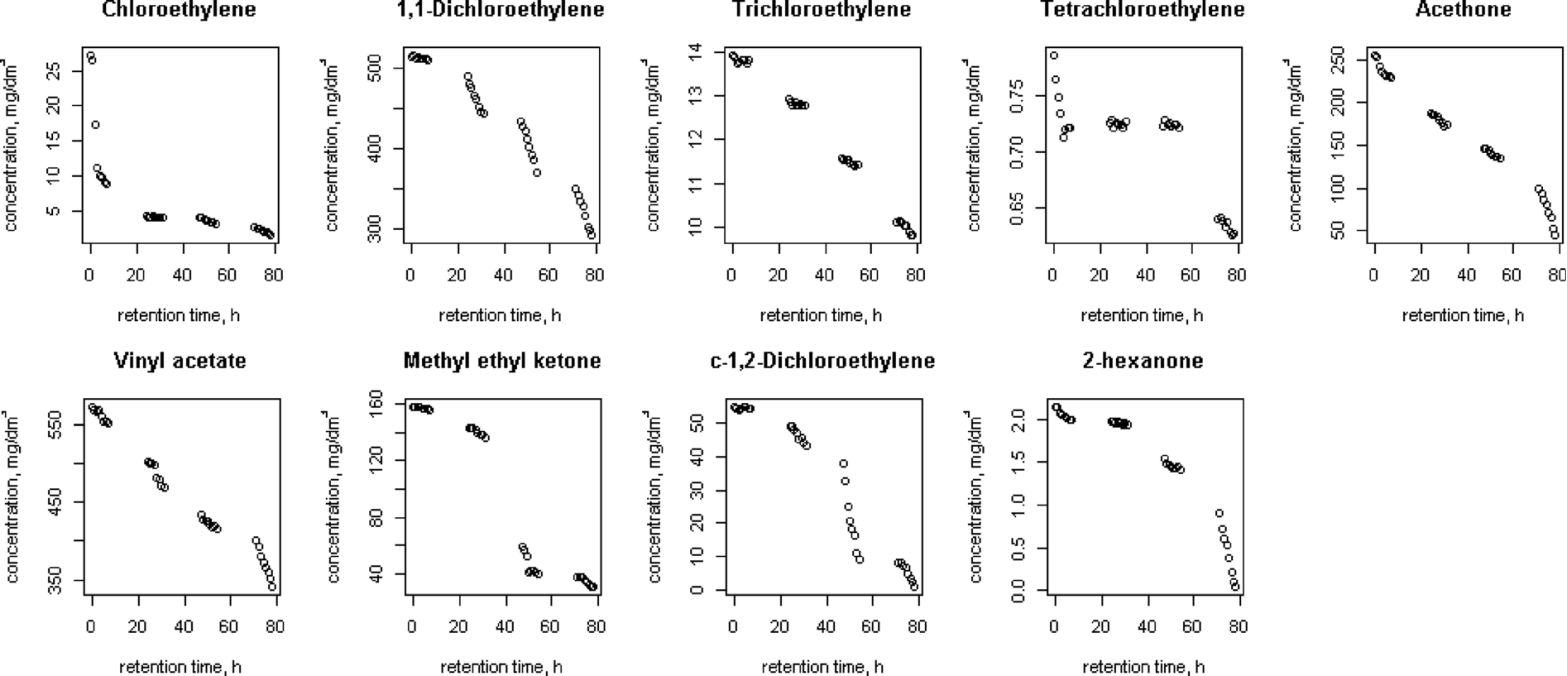
Changes in the concentration of compounds during 78 h of detention in a Tedlar^®^ bags; series 5

**Fig. 6:**
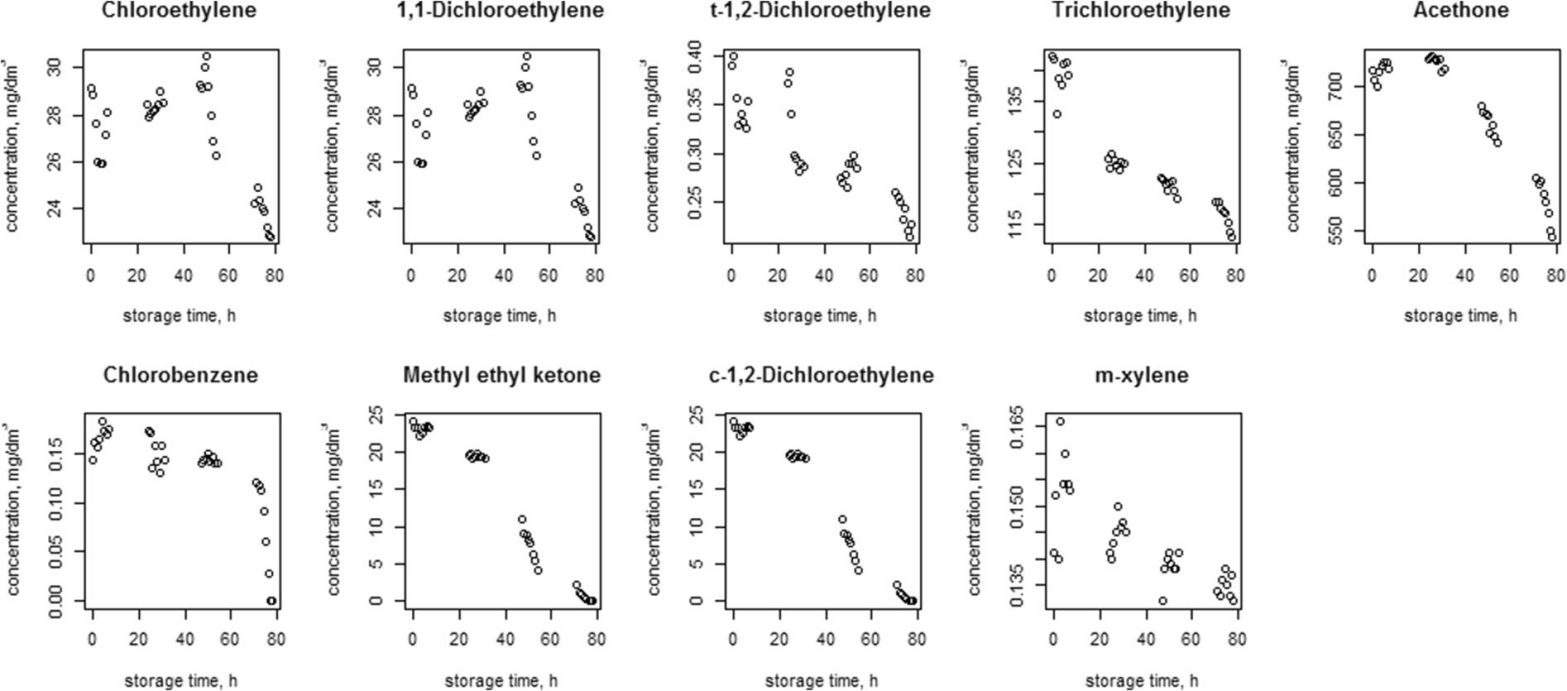
Changes in the concentration of compounds during 78 h of detention in a Tedlar^®^ bags; series 6

Figures 1, 2, 3, 4, 5 and 6 shows changes in the concentration of various compounds in six gas mixtures during 78 h of detention in a Tedlar^®^ bags.

Shapiro-Wilk (SW) as well as the Kolmogorov-Smirnov and Lillefors normality tests were performed for the distribution of each variable, which is the concentration of the compound. Significant values (*p* > 0.05) of the coefficient *p* in SW test were observed for series 1: vinyl acetate (*p* = 0.09), c-1,2 dichloroethylene 1 (*p* = 0.10), t-1.2 -dichloroethylene (*p* = 0.74), series 3: acetone (*p* = 0.07), carbon disulfide (*p* = 0.06), series 4: C, 1,2-dichloroethylene (*p* = 0.11), series 5: acetone (*p* = 0.14) and series 6: t-1,2-dichloroethylene (*p* = 0.30). In other 41 cases, the hypothesis of normal distribution has to be rejected, so it was concluded that non-parametric tests should be applied to the whole population.

Table 2 consists the average rate of change over the considered period (till 30 h, after 30 h and whole period) for each of the compounds, expressed by the difference between the chain index and the one:

**Table 2:** The average rate of loss of compounds concentration

		act	ben	brm	cs2	clb	cle	1 cl	c12	t12	2hx	mek	3et	4ce	vac	vcl	mxy
$\overline{l_g}$	Y_n_ ≤ 30 h	0.10	0.36	0.65	1.52	*1.67*	0.40	0.15	0.37	0.36	0.33	0.02	0.07	0.30	1.64	3.60	*0.24*
		0.71	0.26	–	–	*0.34*	1.15	2.50	1.22	1.04	–	0	0.29	–	1.46	–	*0.14*
		1.54	2.00	–	–	–	0.91	1.29	1.26	–	–	1.70	0.44	–	1.11	–	–
		1.32	–	–	–	–	6.36	0.32	0.71	–	–	0.94	–	–	0.67	–	–
		0.01	–	–	–	–	0.01	0.48	0.74	–	–	0.45	–	–	–	–	–
		–	–	–	–	–	–	0.35	2.26	–	–	0.77	–	–	–	–	–
	Y_n_ ≥ 30 h	6.97	100	*0.94*	6.26	100	8.38	0.77	0.61	100	13.0	100	0.86	0.48	8.05	100	0.55
		100	1.17	–	–	100	18.50	100	100	0.83		100	0.91	–	100	–	0.37
		6.20	6.45	–	–	–	4.27	4.87	9.06	–	–	5.80	0.35	–	1.19	–	–
		4.59	–	–	–	–	3.54	2.75	1.79	–	–	2.62	–	–	1.12	–	–
		0.94	–	–	–	–	0.83	1.46	13.0	–	–	5.03	–	–	–	–	–
		–	–	–	–	–	–	1.96	8.18	–	–	100	–	–	–	–	–
	Y_n_ = 78 h	2.65	100	*0.11*	2.97	100	3.39	0.35	0.37	100	5.24	100	0.35	0.29	3.71	100	0.12
		100	0.54	–	–	100	7.82	100	100	0.70		100	0.45	–	100	–	0.09
		2.34	3.22	–	–	–	1.97	2.34	3.98	–	–	2.86	0.30	–	0.87	–	–
		2.25	–	–	–	–	3.76	1.16	0.94	–	–	1.35	–	–	0.68	–	–
		0.36	–	–	–	–	0.32	0.73	5.37	–	–	2.09	–	–	–	–	–
		–	–	–	–	–	–	0.87	3.99	–	–	100	–	–	–	–	–

Italics indicate concentration growth. Abbrev. as in Table 1

$$ \overline{l_g}=\sqrt[n-1]{\frac{Y_n}{Y_1}}-1 $$

For some of compounds, 100 % decrease of concentration has been noticed within 78 h of holding in the Tedlar^®^ bag. Concentration of those compounds has dropped to zero in the last 48 h; in the first 30 h noticed decrease was: 0.36 % (benzene), 0.71 % (acetone), 2.50 % (1,1-dichloroethylene), 1.22 % (c-1,2-dichloroethylene), 0.36 % (t-1,2-dichloroethylene), 0.02, 0 and 0.77 % (methyl ethyl ketone), 3.60 % (vinyl chloride). The average rate of loss for chlorobenzene during last 48 of 78 h holding in Tedlar^®^ bag was 100 % in two series, but within the first 30 h, an increase of the concentration (1.67 and 0.34 %) had been noticed. Also, increase of concentration had been observed in bromomethane in last 48 h (0.94 %) and m-xylene in first 30 h (0.24 and 0.14 %). In other cases, the average rate of loss was from 0.01 to 2.50 % for first 30 h and from 0.35 to 18.50 % during last 48 h of examination.

**Fig. 7:**
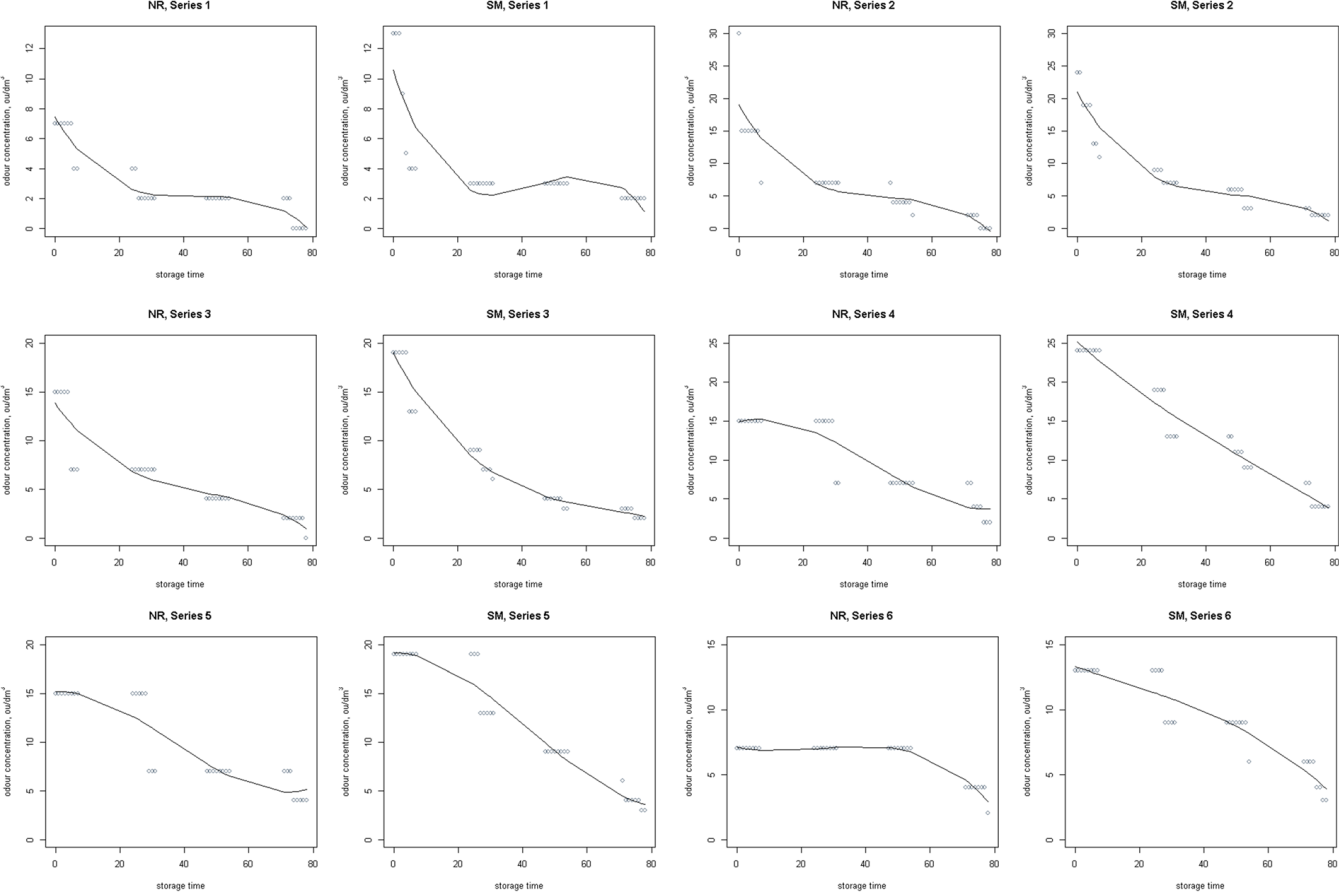
Changes in the odour concentration of various compounds in six gas mixtures during 78 h of detention in a Tedlar^®^ bags, measured by Nasal Ranger and Scentroid SM-100 olfactometers. *X axis* storage time, h; *Y axis* odour concentration, ou/dm^3^

**Fig. 8:**
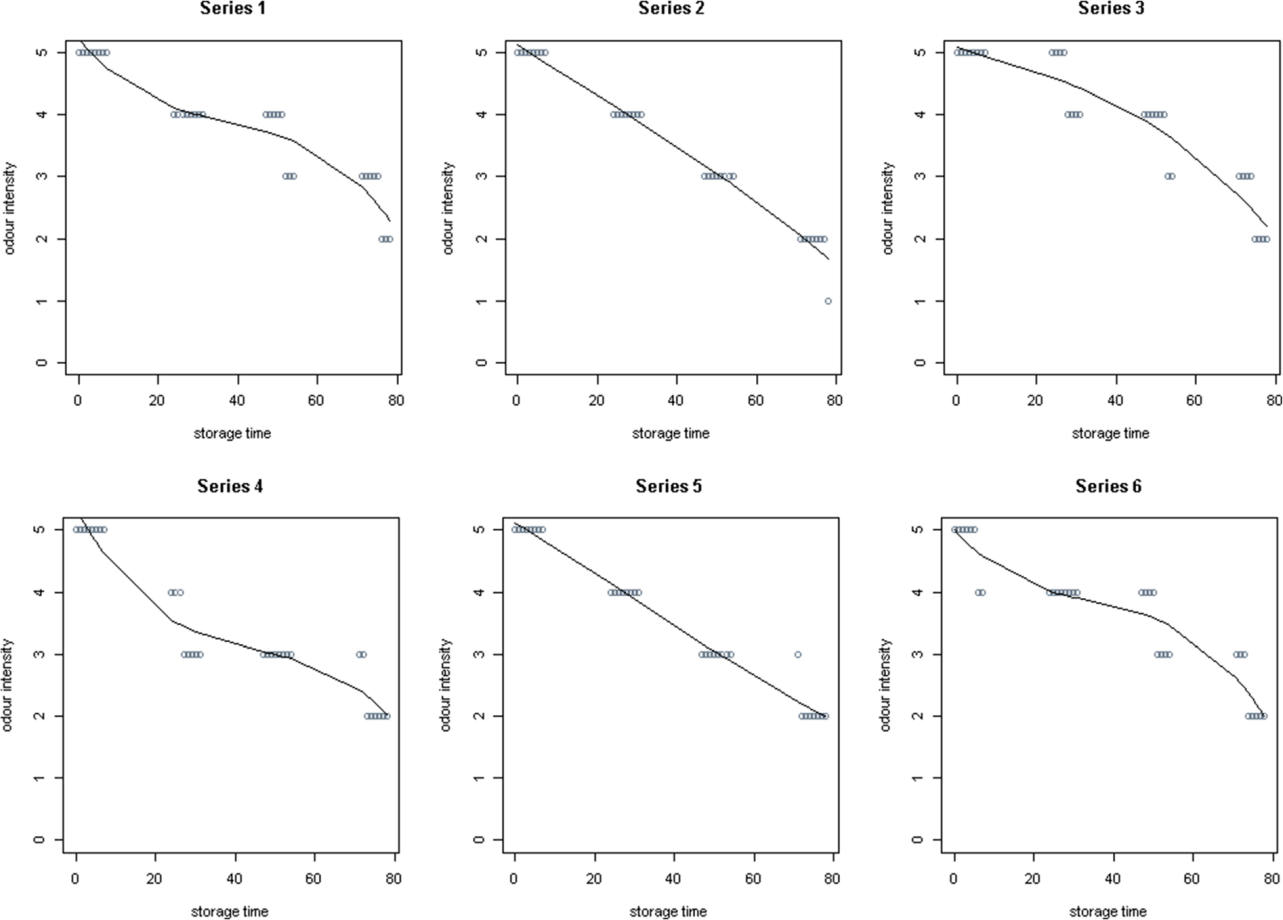
Changes in the odour intensity of various compounds in six gas mixtures during 78 h of detention in a Tedlar^®^ bags. *X axis* storage time, h; *Y axis* odour intensity

Figure 7 shows changes in the odour concentration of various compounds in six gas mixtures during 78 h of detention in a Tedlar^®^ bags, measured by Nasal Ranger and Scentroid SM-100 olfactometers. Moreover, on Fig. 8 odour intensity determined on a scale from 0 (no odour) to 6 (extremely strong) was shown.

For the analysis of the obtained results, the non-parametric regression were used, in which not assumed the knowledge of the analytical form of the relationship between the explanatory variables and the dependent variable as well as knowledge of random component’s distribution in the model. Due to the fact that these tests were designed to solve the task of regression when even some of the classical assumptions of classical are not met, the non-parametric methods often have much greater flexibility and accuracy.

Figures 7 and 8 shown chart of spline functions for the 3°of freedom (basis functions). To build regression models, spline curve method were used (the model for one explanatory variable *h* and the dependent variable, which constituted the odour concentration and odour intensity *i* values). This method is considered as an additive model (Hastie et al. 2009; Trzęsiok 2005):

$$ Y=f(X)={\alpha}_0+{\displaystyle \sum_{k=1}^K}{\alpha}_k{f}_k(X) $$

where *X* is the explanatory variable and *Y* is the dependent variable. Subject matter of variable *X* is divided into *K* disjoint intervals using an ordered set of points (nodes). To overcome the drawback associated with the discontinuity of the function *f*, degree polynomials *fk* was increased and imposed on them the appropriate continuity conditions in nodes. In order to obtain a higher degree of smoothness of the function *f*, also the continuity conditions for the corresponding derivatives were introduced to achieve the spline function of M-order. The number of its degrees of freedom *df* is equal to the number of basis functions. The most widely used are third-order spline functions (Walesiak and Gatnar 2013)—in the present case was also examined *df* = 5, 15 and 35, but the most complete fit was obtained for *df* = 3.

As in case of compounds concentration, the average rate of change of odour concentration and intensivity over the considered period (till 30 h, after 30 h and whole period) had been calculated. Decreasing of odour concentration measured by Nasal Ranger (NR) in all series was between 0.00 and 4.98 % (mean 2.82 %, median 2.59 %) till 30 h, between 1.91 and 100 % (mean 51.73 %, median 52.11 %) in the last 48 h of test and between 1.61 and 100 % (mean 50.98 %, median 51.29 %) in 78 h. In case of odour concentration measured by SM, those values were, respectively, 1.26–4.93 % (mean 2.85 %, median 2.74 %), 1.39–4.93 % (mean 3.75 %, median 4.11 %) and 2.40–3.18 % (mean 2.50 %, median 2.39 %). Values of average rate of intensity decreasing were, respectively, 0.77–1.75 % (mean 0.93 %, median 0.77 %), 2.36–4.67 % (mean 2.58 %, median 2.36 %) and 1.18–2.07 % (mean 1.33 %, median 1.18 %).

Strength of association of the correlation between the values of odour concentration designated by SM and NR, as well as odour intensity and the concentration of individual compounds in six series of research, was determined on the basis of Spearman’s rank correlation coefficient. The ranked correlation always assumes values in the range [−1, +1]. The interpretation is similar to the classic Pearson correlation coefficient, but based on the Pearson coefficient, a linear relationship between the variables is measured and all other compounds are treated as linear dependence impaired, wherein the ranked correlation shows any monotonic relation (also non-linear). To interpret, the following scale was used: *r* = 0, correlation does not exist, the lack of correlation, the variables are uncorrelated; 0 < | *r* | <0.3, weak level of interdependence; 0.3 ≤ | *y* | <0.5, the average degree of interdependence; 0.5 ≤ | *y* | <0.7, a significant degree of interdependence; 0.7 ≤ | *y* | <0.9, a high degree of interdependence; | *r* | ≥ 0.9, very high degree of correlation; | *r* | = 1, total correlation (accuracy); functional relationship between the considered features (Górecki 2011). Square of the correlation coefficient, *R*^2^, is the coefficient of determination, indicating which part of the dependent variable is explained by changes in explanatory variable.

Rank (order) correlation coefficient was calculated by the formula:

$$ {r}_S=1-\frac{6{\displaystyle {\sum}_{i=1}^n}{\left({X}_i-{Y}_i\right)}^2}{n\left({n}^2-1\right)} $$

To test the significance of the Spearman correlation coefficient, the test statistic was applied, which with the truth of the null hypothesis has distribution *t*(*n*-*2*):

$$ T=\frac{r_S}{\sqrt{1-{r}_S^2}}\sqrt{n-2} $$

**Fig. 9:**
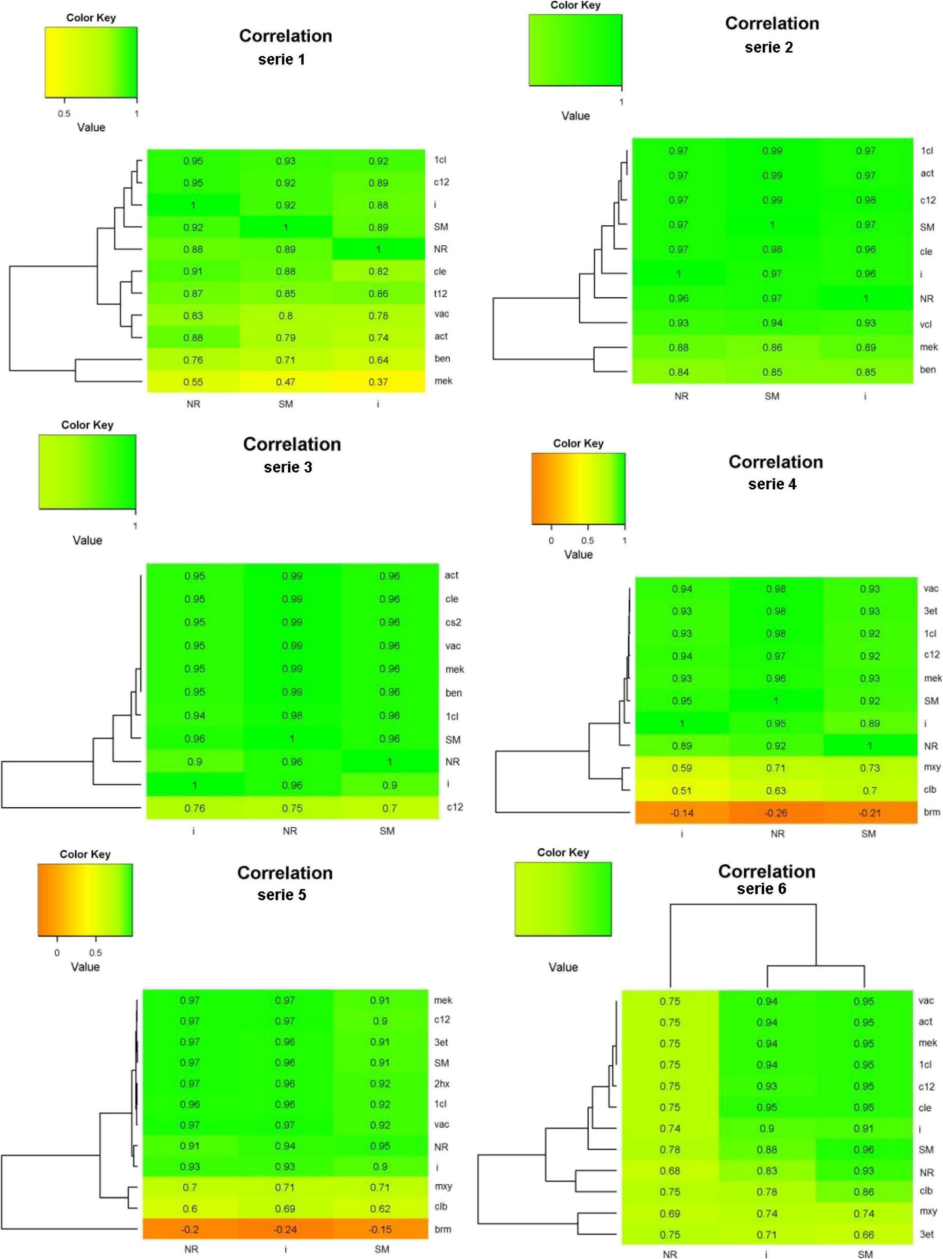
Heatmaps with dendrograms of the Spearman correlation coefficient between all variables in series 1–6. *NR* odour concentration measured by Nasal Ranger, *I* intensity, *SM* odour concentration measured by Scentroid SM-100. Compound abbreviations as in Table 1

Figure 9 contains heatmaps with dendrograms of the Spearman correlation coefficient between all variables in 6 series.

In case of series 1, correlation coefficients for intensity and compounds concentration were from 0.55 (mek) to 0.95 (1,1-dichloroethylene and 1,2-dichloroethylene), in other cases were high and very high, from 0.76 to 0.91. Also for SM and NR, lowest correlation coefficient was reported to mek concentration (0.47 and 0.37); in other cases, it amounted, respectively, 0.71–0.93 and from 0.64 (benzene) to 0.92. In series 2, correlation coefficients for intensity and compounds concentration were from 0.84 (benzene) to 0.97, and SM, NR for compounds concentration, respectively, from 0.85 (benzene) to 0.99 and from 0.85 (benzene) to 0.98. In series 3, those results were, respectively, 0.76–0.95, 0.75–0.99 and 0.70–0.96. The lowest values were conducted in case of c-1.2-dichloroethylene. In series 4, results were as follows: −0.14–0.94, −0.26–0.98, and −0.21–0.93. Negative, weak degree of correlation was observed for bromomethane. In series 5, the values were as follows: 0.60–0.97, 0.63–0.97 and 0.58–0.95. The lowest values were conducted in case of tetrachloroethylene. In series 3, following values were conducted: 0.52–0.93, 0.45–0.95 and 0.69 (m-xylene)-0.78. In case of intensity and SM, the lowest values were noticed in concentration of chloroethene. All of the above results, except bromomethane concentration and intensity, NR as well as SM (*p*, respectively, 0.45, 0.15, 0.25) were statistically significant (*p* <0.05).

Different authors studied influence of different bag materials to sample degradation. Zarra et al. (2012) done a critical evaluation of relation to the same odour source with a comparison of sampling bag materials and intervals of time elapsed between the sampling and analysis phase (3, 7, 14, 30 and 48 h). They proved that inter alia, ammonia and hydrogen sulfide easily escape from Nalophan sample bags, so the 30-h expiration period on all samples could not be performed. They have shown that in Teflon bags are the most stable, while Nalophan bags are less reliable. Hansen et al. (2011) have shown that the concentrations of carboxylic acids, phenols and indoles decreased by 50 to >99 % during the 24 h of storage in Tedlar and Nalophan bags. The concentration of hydrogen sulfide decreased by approximately 30 % during the 24 h of storage. According to Coyne et al. (2003), in 24 h, Tedlar^®^ bags have losses of less than 5 % on sulfur compounds. However, ammonia—40 % in 24 h and 60 % in 30 h—and phenol—50 % in 24 h—still escape from the Tedlar^®^ sample bag. van Harreveld (2003) concluded that Nalophan film bags performed significantly better than metalized Cali-Bond layered film as a bag material. The odour concentration of samples in Nalophan bags remained relatively stable between 4 and 12 h after sampling. After 30 h, decay to about half the initial concentration, as measured at 4 h, was observed. Particle removal during sampling caused the odour concentration in the bags to be reduced by approximately 20 %. Mochalski et al. (2009) suggested analyzing the breath VSCs within 6 h after sampling. Flexfoil bags were found to be the best choice for the VSCs storage up to 24 h (recovery about 90 % with the exception of DMS). For shorter storing times (6–8 h), transparent Tedlar is a good alternative for Flexfoil (losses up to 10 %). Bakhtari (2014) studied degradation rate of odour concentration in Nalophan, Tedlar^®^, and PTFE bags and have shown that Nalophan has the highest odour decay followed by Tedlar^®^, and PTFE has the best odour preservation. For waste water treatment, plant odour samples have high degradation in both Nalophan (60 % in 24 h) and Tedlar^®^ (35 %) but far better in PTFE sample bags (23 %). Kim et al. (2012) compared the stability of polyester aluminum (PEA) and Tedlar^®^ bags for gaseous VOC sampling. Eight VOC standards: benzene, toluene, p-xylene, styrene, methyl ethyl ketone, methyl isobutyl ketone, butyl acetate and isobutyl alcohol, were placed into each bag at storage times of 0, 2 and 3 days prior to analyses by gas chromatography/mass spectrometry (GC/MS). They concluded, that although the Tedlar^®^ showed fairly stable relative recovery values for most VOCs (i.e., about 89 % after 2 days), they decreased to 73 % after 3 days. For PEA, those values of all target VOCs were recorded with improved recoveries (relative to Tedlar^®^), i.e. 93 % (*t* = 2 days) and 88 % (*t* = 3 days). Hsieh et al. (2003) examined 56 volatile organic compounds, known to be ozone precursors, which were stored in three media (SUMMA, Silocan canisters and Tedlar bags) to evaluate their stability in these storage media. After a 7-day storage period, 87 % of alkenes could be recovered from canister storage, and 82 % were recovered from Tedlar bag storage. Isoprene, a major component in biogenic VOCs, exhibited a recovery rate of only 75 ± 8 % after storage for 7 days in canisters and Tedlar bags. The VOCs stored in Tedlar bags had a lower recovery than those stored in canisters. Coyne et al. (2003) evaluated SamplePro^®^ FlexFilm (proprietary material), FlexFoil^®^ PLUS, FluoroFilm FEP and Tedlar^®^ for effectiveness in holding 32 volatile organic compounds (VOCs). Based on the study data, the best film choices for VOC collection are SamplePro FlexFilm, FlexFoil PLUS and Tedlar. FlexFoil PLUS is an optimal alternative for the collection of sulfur compounds. SamplePro FlexFilm, FlexFoil PLUS and Tedlar are the best film alternatives for CO, CO_2_ and methane. FlexFoil PLUS is the best choice for hydrogen. None of the films tested are recommended for nitrogen dioxide. All films tested may be used for sulfur hexafluoride with good results (Coyne et al. 2003). The VOCs were tested by injecting pure, known volumes of the test analyte into the bag filled with nitrogen, and analysis was performed on day 0, day 1 and day 2. In several cases, researchers achieved increase of compound concentration during the 2-day period. Laor et al. (2010) shown that the storage had the greatest impact on coffe odour (both in Tedlar and Nalophan) with average losses by factors of 4–5, and for manure odour in Tedlar bags with average losses by factors around 6 (storage time 24 h). Those authors achieved opposite effects for sewage odour, with losses by factor around 2 in Tedlar, but increase of odour by factors around 3 in Nalophan. Akdeniz et al. (2011) studied assessment of the impact of hydrogen sulfide, total reduced sulfur (TRS), ammonia, methane and nitrous oxide stability in Tedlar and FlexFoil bags. Percent recoveries from FlexFoil bags ranged from 75 to 99.5 % for all gases and concentrations except for TRS at high concentrations. For TRS at high concentrations, percent recovery from FlexFoil bags was 68.8 %. No gas desorption or permeation was observed when using new FlexFoil bags. Gnosh et al. (2011) examined the patterns of VOCs released from Tedlar bags that were once used for the collection under strong source activities and attempted to account for the possible bias associated with the repetitive use of Tedlar bags. The overall results of their study consistently indicate that polar compounds can be subject to negative bias more significantly (due to their affinity on the Tedlar bags) than nonpolar compounds (other than toluene). Those authors mentioned that caution on the nature and extent of bias when sampling and analyzing VOCs based on grab sampling methods like Tedlar bag sampler should be taken (Gnosh et al. 2011). Traube et al. (2006) study was conducted to determine if Tedlar bags affect the integrity of sampled air from animal operations—air samples were collected simultaneously in both Tedlar bags and Tenax thermal desorption tubes. After 24 h of storage, recovery of C3-C6 volatile fatty acids (VFA) averaged 64 %, 4-methylphenol and 4-ethylphenol averaged 10 %, and indole and 3-methylindole were below the detection limits of GC-MS-O. The odour activity value (OAV) of grab samples collected in Tedlar bags were 33 to 65 % lower following 24 h of storage. These results indicate that significant odorant bias occurs when using Tedlar bags for the sampling of odours from animal production facilities (Traube et al. 2006). Sironi proved that the ammonia losses from the NalophanTM sampling bag always turned out to be significant; for instance, in the case of a bag with a surface of 2580 cm^2^ filled with 6000 cm^3^ of gas, the percent ammonia loss after 26 h was 37 %. This value is not negligible especially considering that the European Norm EN 13725:2003 allows a maximum storage time of 30 h, thus assuming that the sampled mixture remains almost unalerted for 30 h (Sironi et al. 2014). From the results of a comparative study between Tedlar bag and Flek polyester bag, it was found that Flek polyester bag was more consistent than Tedlar bag. Tiwari et al. (2010) measured the change in total hydrocarbon standard concentration using these two different types of bags with respect to time (0, 1, 2, 4 and 8 h). The comparative study illustrates that the difference in results (from 0 to 8 h) was observed high (about 640 ppb) for Tedlar bag, while less difference was observed (30 ppb) for Flek polyester bag.

## Summary and Conclusions

According to the main goal of the study, determination of the uncertainty during storage of different samples up to 24 h had been done. For some of compounds listed below, 100 % decrease of concentration has been noticed within 78 h of holding in the Tedlar^®^ bag. Concentration of the benzene, acetone, 1,1-dichloroethylene, c-1,2-dichloroethylene, t-1,2-dichloroethylene, methyl ethyl ketone and vinyl chloride. Increase of the concentration of chlorobenzene and m-xylene during the first 30 h as well as increase bromomethane concentration in the last 48 h had been noticed. In other cases, the average rate of loss was from 0.01 to 2.50 % for the first 30 h and from 0.35 to 18.50 % during the last 48 h of examination. Correlation coefficients for compound concentrations and intensity, odour concentration obtained by SM-100 as well as NR were, respectively, from −0.14 to 0.97, from −0.26 to 0.99 and from −0.21 to 0.98. Negative, weak degree of correlation was observed for bromomethane, but those values were not statistically significant. The second goal was to determine the usability of NR and SM-100 for those measurements and check obtained differences. Decreasing of odour concentration measured by NR in all series was between 0.00 and 4.98 % till 30 h, between 1.91 and 100 % in the last 48 h of test and between 1.61 and 100 % in 78 h. In case of odour concentration measured by SM, those values were, respectively, 1.26–4.93 % (median 2.74 %), 1.39–4.93 % (median 4.11 %) and 2.40–3.18 % (median 2.39 %). Differences in the results obtained using two olfactometers due to the accuracy of the determination of odour concentration—SM-100 has a lower determination threshold than NR. Values of average rate of intensity decreasing were, respectively, 0.77–1.75 % (median 0.77 %), 2.36–4.67 % (median 2.36 %) and 1.18–2.07 % (median 1.18 %). There are many techniques that can be deployed in order to minimize sample degradation; for example, nitrogen-based pre-dilution and sealed transportation vessels (Bakhtari 2014). A variety of different sampling strategies and sorbent media have been developed to address specific applications. Key sorbent-based examples include the following: active sampling onto tubes packed with one or more sorbents held at ambient temperature; diffusive sampling onto sorbent tubes/cartridges; on-line sampling of air/gas streams into cooled sorbent traps; and transfer of air samples from containers into cooled sorbent focusing traps (Woolfenden 2010). In accordance with the European Standard “Air quality—Determination of odour concentration by dynamic olfactometry” (EN 13725; CEN 2003), and sampling bags such as Tedlar^®^ or Nalophan^®^ are considered appropriate for odour examinations. Sample storage up to 30 h is allowed before measurement (Laor et al. 2010). Bakhtari wrote that German standard VDI3880, and possible the soon to be revised EN13725 standard, limit sample storage to 6 h unless it can be shown that the sample degradation is within acceptable limit. Some samples can degrade by an order of ten magnitude in a span of less than 24 h. Sample degradation is based on a number of factors including: sample composition, pre-dilution to minimize condensation, sample bag material, transportation method, and duration of storage (Bakhtari 2014). In accordance with the European Standard “Air quality—Determination of odour concentration by dynamic olfactometry” (EN 13725; CEN 2003), sampling bags such as Tedlar^®^ or Nalophan^®^ are considered appropriate for odour examinations. According to those examinations, some alternatives should be used. Also, it should be clearly defined and regulated which bags are suitable for different odour sources. For examined samples, probably better than Tedlar^®^ or Nalophan^®^ would be PTFE which had a very good stability i.e. for VOCs and is recommended for any petrochemical products.
